# FEASIBILITY OF CARRES MODULES TO REDUCE POTENTIALLY AVOIDABLE HOSPITALIZATIONS IN PERSONS WITH COGNITIVE DEFICITS

**DOI:** 10.1093/geroni/igz038.1063

**Published:** 2019-11-08

**Authors:** Victoria Steiner, Linda Pierce, Carol Bryan

**Affiliations:** University of Toledo, Toledo, Ohio, United States

## Abstract

Family caregiving is an essential, yet understudied, factor that can hasten, delay, or prevent hospital readmissions in individuals with cognitive deficits. This 3-month feasibility study examined 18 Internet-based educational CARREs (Communicate, Assist, Recognize & Report Events) Modules for family caregivers that address care recipients’ potentially avoidable hospitalization (PAH) conditions, e.g. UTI. This study determined: 1) caregivers’ perceptions about the use of the CARREs Modules, 2) caregivers’ self-reported value of the Modules, and 3) potential outcomes for caregivers and care recipients. Community-dwelling family caregivers were recruited from local support/education programs and assigned 6-8 Modules based on their care recipients’ needs. Links to online surveys were emailed at baseline, and 30 and 90 days post-enrollment. Descriptive statistics were performed on these data. Twenty potential subjects were screened but five were ineligible and three refused to participate. The remaining 12 subjects were primarily White females caring for a husband or parent. Subjects completed all the Modules they were assigned and did not experience any difficulties answering the survey questions. Subjects reported completing the Modules at least moderately increased their knowledge (67%), not being burdened by completing the Modules (67%), and very likely to participate again (58%). Many subjects stated the Modules taught them new things about preventing hospital readmissions in their care recipient (75%) and improved their well-being as a caregiver (83%). In collaboration with a home care agency, the investigators plan to implement and test a sustainable, “real-world” educational intervention incorporating the CARREs Modules that reaches a wide audience of family caregivers.

